# (8a*S*)-7,8,8a,9-Tetra­hydro­thieno[3,2-*f*]indolizin-6(4*H*)-one

**DOI:** 10.1107/S1600536809007405

**Published:** 2009-03-06

**Authors:** Ľubomír Švorc, Viktor Vrábel, Jozef Kožíšek, Štefan Marchalín, Peter Šafář

**Affiliations:** a Institute of Analytical Chemistry, Faculty of Chemical and Food Technology, Slovak Technical University, Radlinského 9, SK-812 37 Bratislava, Slovak Republic; b Institute of Physical Chemistry and Chemical Physics, Faculty of Chemical and Food Technology, Slovak Technical University, Radlinského 9, Bratislava, Slovak Republic 81237; c Institute of Organic Chemistry, Catalysis and Petrochemistry, Faculty of Chemical and Food Technology, Slovak Technical University, Radlinského 9, SK-812 37 Bratislava, Slovak Republic

## Abstract

In the mol­ecular structure of the title compound, C_10_H_11_NOS, the central six-membered ring of the indolizine unit adopts an envelope conformation, the maximum deviations from the mean plane of the ring being 0.533 (2) Å. The fused thieno ring is nearly coplanar [mean deviation = 0.007 (2) Å]. The conformation of the fused oxopyrrolidine ring is close to that of a flat-envelope, with a maximum deviation of 0.339 (3) Å. The crystal structure is stabilized by C—H⋯O hydrogen bonds.

## Related literature

For general applications of indolizine derivatives, see: Brandi *et al.* (1995 ▶); Campagna *et al.* (1990 ▶); Couture *et al.* (2000 ▶); Gubin *et al.* (1992 ▶); Gundersen *et al.* (2003 ▶); Gupta *et al.* (2003 ▶); Hema *et al.* (2003 ▶); Hempel *et al.* (1993 ▶); Jorgensen *et al.* (2000 ▶); Malonne *et al.* (1998 ▶); Marchalín *et al.* (2008 ▶); Medda *et al.* (2003 ▶); Nardelli (1983 ▶); Pearson & Guo (2001 ▶); Poty *et al.* (1994 ▶); Rosseels *et al.* (1982 ▶); Sonnet *et al.* (2000 ▶); Vlahovici *et al.* (2002 ▶); Vrábel *et al.* (2004 ▶); Švorc *et al.* (2007 ▶). For bond-length data, see: Brown & Corbridge (1954 ▶); Pedersen (1967 ▶).
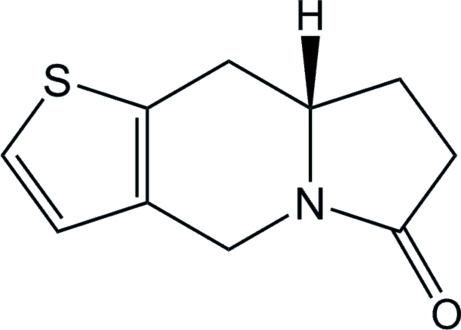


## Experimental

### 

#### Crystal data

C_10_H_11_NOS
*M* *_r_* = 193.26
Triclinic, 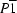

*a* = 6.37912 (16) Å
*b* = 8.3654 (3) Å
*c* = 9.0715 (3) Å
α = 84.180 (3)°
β = 78.611 (2)°
γ = 76.174 (3)°
*V* = 460.06 (3) Å^3^
*Z* = 2
Mo *K*α radiation
μ = 0.31 mm^−1^
*T* = 298 K
0.42 × 0.32 × 0.14 mm

#### Data collection

Oxford Diffraction Gemini R CCD diffractometer
Absorption correction: analytical (Clark & Reid, 1995 ▶) *T*_min_ = 0.824, *T*_max_ = 0.928
20197 measured reflections
2348 independent reflections
1918 reflections with *I* > 2σ(*I*)
*R*_int_ = 0.018

#### Refinement

*R*[*F* ^2^ > 2σ(*F* ^2^)] = 0.040
*wR*(*F* ^2^) = 0.118
*S* = 1.06
2348 reflections
118 parameters
H-atom parameters constrained
Δρ_max_ = 0.41 e Å^−3^
Δρ_min_ = −0.21 e Å^−3^

### 

Data collection: *CrysAlis CCD* (Oxford Diffraction, 2006 ▶); cell refinement: *CrysAlis RED* (Oxford Diffraction, 2006 ▶); data reduction: *CrysAlis RED*; program(s) used to solve structure: *SHELXS97* (Sheldrick, 2008 ▶); program(s) used to refine structure: *SHELXL97* (Sheldrick, 2008 ▶); molecular graphics: *DIAMOND* (Brandenburg, 2001 ▶); software used to prepare material for publication: *enCIFer* (Allen *et al.*, 2004 ▶).

## Supplementary Material

Crystal structure: contains datablocks I. DOI: 10.1107/S1600536809007405/bg2239sup1.cif

Structure factors: contains datablocks I. DOI: 10.1107/S1600536809007405/bg2239Isup2.hkl

Additional supplementary materials:  crystallographic information; 3D view; checkCIF report

## Figures and Tables

**Table 1:** Hydrogen-bond geometry (Å, °)

*D*—H⋯*A*	*D*—H	H⋯*A*	*D*⋯*A*	*D*—H⋯*A*
C9—H9*A*⋯O1^i^	0.93	2.60	3.379 (2)	142

Symmetry code: (i) 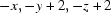.

**Fig. 1.**
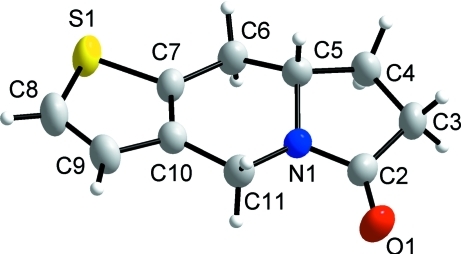
Molecular structure of (I) with the atomic numbering scheme. Displacement ellipsoids are drawn at the 50% probability level (Brandenburg, 2001).

## supplementary crystallographic information

### Comment

Indolizines, the nitrogen containing heterocyclic systems, are widely distributed in nature; in particular, indolizine derivatives are an important class of heterocyclic bioactive compounds with a wide range of applications, such as pharmaceutical drugs, potential central nervous system depressants, calcium entry blockers, cardiovascular agents, spectral sensitizers and novel dyes (Gubin *et al.*, 1992; Gupta *et al.*, 2003; Poty *et al.*, 1994; Hema *et al.*, 2003). Polycyclic indolizine derivatives have been found to have high-efficiency long-wavelength fluorescence quantum yield (Vlahovici *et al.*, 2002). Several polyhydroxylated indolizines are interesting as inhibitors of glycosides (Hempel *et al.*, 1993; Brandi *et al.*, 1995). They have also been tested as antimycobacterial agents against mycobacterial tuberculosis (Gundersen *et al.*, 2003), for the treatment of angina pectoris (Rosseels *et al.*, 1982), aromatase inhibitory (Sonnet *et al.*, 2000), antiinflammatory (Malonne *et al.*, 1998), antiviral (Medda *et al.*, 2003), analgestic (Campagna *et al.*, 1990) and antitumor (Pearson & Guo, 2001) activities. The other well known pharmacological applications associated with this ring compounds are well documented in the literature (Couture *et al.*, 2000; Jorgensen *et al.*, 2000). These findings had led to a spate of synthetic and structural studies of various indolizine analogues.

Based on these facts and in continutation of our interest in developing simple and efficient routes for the synthesis of novel indolizine derivatives, we report here the synthesis, molecular and crystal structure of the title compound, (I). The absolute configuration is known from the synthesis and is depicted in the scheme and Figure 1. The expected stereochemistry of atom C5 was confirmed as *S*. The central N-heterocyclic ring is not planar and adopts an envelope conformation (Nardelli, 1983). A calculation of least-squares planes shows that this ring is puckered in such a manner that the five atoms N1, C11, C10, C7 and C6 are coplanar to within 0.032 (3) Å, while atom C5 is displaced from this plane with out-of-plane displacement of 0.533 (2) Å. The fused thieno ring is nearly coplanar [mean deviation = 0.007 (2) Å]. The oxopyrrolidine ring attached to the indolizine ring system has flat-envelope conformation with atom C5 on the flap. The deviation of atom C5 from the mean plane of the remaining four atoms N1/C2/C3/C4 is 0.339 (3) Å. The N1—C5 and N1—C11 bonds are approximately equivalent and both are much longer than the N1—C2 bond. Moreover, the N1 atom is *sp*^2^ hybridized, as evidenced by the sum of the valence angles around it [356.1 (1)°]. These data are consistent with conjugation of the lone-pair electrons on N1 with the adjacent carbonyl and agree with literature values for simple amides (Brown & Corbridge, 1954; Pedersen, 1967). The bond length of the carbonyl group C2=O1 is 1.224 (2) Å, respectively, is somewhat longer than typical carbonyl bonds.  This may be due to the fact that atom O1 participates in intermolecular C–H···O hydrogen bonds with atom C9 (Table 2). The bond lengths and angles in the molecule are comparable with those in a related structure (Vrábel *et al.*,  2004,Švorc *et al.*, 2007).

### Experimental

Triethylsilane (2.4 ml, 15 mmol) was added to a stirred solution of alcohol (2.1 g, 10 mmol) in trifluoroacetic acid (20 ml) at 0 °C, and the resulting yellow solution was stirred at rt for 2 h. The reaction mixture was concentrated *in vacuo*, diluted with water (50 ml), made alkaline with 10% Na_2_CO_3_, and then extracted with dichloromethane (3 *x* 50 ml). The extract was washed with water, dried over magnesium sulfate, and concentrated *in vacuo*. The residue was purified by flash chromatography on a silica gel column eluting with dichloromethane. Recrystallization of a solid from cyclohexane gaves amide as colorless crystals (Marchalín *et al.* 2008).

### Refinement

All H atoms were placed in geometrically idealized positions and constrained to ride on their parent atoms, with C—H distances in the range 0.93 - 0.98Å and *U*_iso_ set at 1.2*U*_eq_ of the parent atom. The absolute configuration could not be reliably determined for this compound using Mo radiation, and has been assigned according to the synthesis.

### Crystal data

**Table:**

C_10_H_11_NOS	*Z* = 2
*M**_r_* = 193.26	*F*(000) = 204
Triclinic, *P*1	*D*_x_ = 1.395 Mg m^−^^3^
Hall symbol: -P 1	Mo *K*α radiation, λ = 0.71073 Å
*a* = 6.37912 (16) Å	Cell parameters from 12211 reflections
*b* = 8.3654 (3) Å	θ = 3.3–29.4°
*c* = 9.0715 (3) Å	µ = 0.31 mm^−^^1^
α = 84.180 (3)°	*T* = 298 K
β = 78.611 (2)°	Block, colourless
γ = 76.174 (3)°	0.42 × 0.32 × 0.14 mm
*V* = 460.06 (3) Å^3^	

### Data collection

**Table:**

Oxford Diffraction Gemini R CCD diffractometer	2348 independent reflections
Radiation source: fine-focus sealed tube	1918 reflections with *I* > 2σ(*I*)
graphite	*R*_int_ = 0.018
Detector resolution: 10.4340 pixels mm^-1^	θ_max_ = 29.5°, θ_min_ = 3.3°
Rotation method data acquisition using ω and φ scans	*h* = −8→8
Absorption correction: analytical (Clark & Reid, 1995)	*k* = −11→11
*T*_min_ = 0.824, *T*_max_ = 0.928	*l* = −12→12
20197 measured reflections	

### Refinement

**Table:**

Refinement on *F*^2^	Primary atom site location: structure-invariant direct methods
Least-squares matrix: full	Secondary atom site location: difference Fourier map
*R*[*F*^2^ > 2σ(*F*^2^)] = 0.040	Hydrogen site location: inferred from neighbouring sites
*wR*(*F*^2^) = 0.118	H-atom parameters constrained
*S* = 1.06	*w* = 1/[σ^2^(*F*_o_^2^) + (0.0629*P*)^2^ + 0.1103*P*] where *P* = (*F*_o_^2^ + 2*F*_c_^2^)/3
2348 reflections	(Δ/σ)_max_ < 0.001
118 parameters	Δρ_max_ = 0.41 e Å^−^^3^
0 restraints	Δρ_min_ = −0.21 e Å^−^^3^

### Special details

**Table:**

Experimental. face-indexed (*CrysAlis RED*; Oxford Diffraction, 2006)The absolute configuration could not be reliably determined for this compound using Mo-radiation, and has been assigned according to the synthesis.
Geometry. All e.s.d.'s (except the e.s.d. in the dihedral angle between two l.s. planes) are estimated using the full covariance matrix. The cell e.s.d.'s are taken into account individually in the estimation of e.s.d.'s in distances, angles and torsion angles; correlations between e.s.d.'s in cell parameters are only used when they are defined by crystal symmetry. An approximate (isotropic) treatment of cell e.s.d.'s is used for estimating e.s.d.'s involving l.s. planes.
Refinement. Refinement of *F*^2^ against ALL reflections. The weighted *R*-factor *wR* and goodness of fit *S* are based on *F*^2^, conventional *R*-factors *R* are based on *F*, with *F* set to zero for negative *F*^2^. The threshold expression of *F*^2^ > σ(*F*^2^) is used only for calculating *R*-factors(gt) *etc*. and is not relevant to the choice of reflections for refinement. *R*-factors based on *F*^2^ are statistically about twice as large as those based on *F*, and *R*- factors based on ALL data will be even larger.

### Fractional atomic coordinates and isotropic or equivalent isotropic displacement parameters (Å^2^)

**Table:**

	*x*	*y*	*z*	*U*_iso_*/*U*_eq_	
C2	0.1565 (3)	0.7697 (2)	1.25520 (17)	0.0449 (4)	
C3	0.2584 (3)	0.6972 (2)	1.39075 (18)	0.0519 (4)	
H3B	0.1863	0.6133	1.4449	0.062*	
H3A	0.2485	0.7821	1.4588	0.062*	
C4	0.4980 (3)	0.6222 (2)	1.32515 (17)	0.0482 (4)	
H4B	0.5952	0.6373	1.3899	0.058*	
H4A	0.5196	0.5054	1.3120	0.058*	
C5	0.5387 (2)	0.7177 (2)	1.17316 (16)	0.0411 (3)	
H5A	0.5841	0.8180	1.1880	0.049*	
C6	0.7046 (2)	0.6242 (2)	1.05152 (16)	0.0437 (3)	
H6B	0.8519	0.6205	1.0671	0.052*	
H6A	0.6866	0.5118	1.0551	0.052*	
C7	0.6721 (2)	0.70843 (18)	0.90130 (16)	0.0397 (3)	
C8	0.6992 (3)	0.8129 (2)	0.63501 (19)	0.0548 (4)	
H8A	0.7385	0.8395	0.5328	0.066*	
C9	0.4964 (3)	0.8674 (2)	0.71745 (17)	0.0481 (4)	
H9A	0.3807	0.9362	0.6779	0.058*	
C10	0.4802 (2)	0.80769 (17)	0.87139 (16)	0.0370 (3)	
C11	0.2797 (2)	0.84994 (19)	0.99057 (16)	0.0413 (3)	
H11B	0.2413	0.9680	1.0020	0.050*	
H11A	0.1577	0.8193	0.9603	0.050*	
N1	0.31810 (18)	0.76400 (15)	1.13355 (13)	0.0369 (3)	
O1	−0.03879 (19)	0.82620 (19)	1.25376 (15)	0.0653 (4)	
S1	0.87330 (7)	0.68901 (6)	0.74263 (5)	0.05585 (18)	

### Atomic displacement parameters (Å^2^)

**Table:**

	*U*^11^	*U*^22^	*U*^33^	*U*^12^	*U*^13^	*U*^23^
C2	0.0358 (8)	0.0559 (9)	0.0399 (8)	−0.0084 (6)	−0.0014 (6)	−0.0036 (6)
C3	0.0459 (9)	0.0707 (11)	0.0334 (7)	−0.0092 (8)	0.0004 (6)	−0.0006 (7)
C4	0.0413 (8)	0.0636 (10)	0.0350 (7)	−0.0049 (7)	−0.0079 (6)	0.0042 (7)
C5	0.0320 (7)	0.0511 (8)	0.0376 (7)	−0.0046 (6)	−0.0068 (6)	−0.0004 (6)
C6	0.0346 (7)	0.0518 (8)	0.0373 (7)	0.0008 (6)	−0.0045 (6)	0.0026 (6)
C7	0.0367 (7)	0.0445 (8)	0.0326 (7)	−0.0041 (6)	−0.0008 (5)	−0.0010 (5)
C8	0.0668 (11)	0.0585 (10)	0.0321 (7)	−0.0093 (8)	−0.0013 (7)	0.0036 (7)
C9	0.0583 (10)	0.0458 (8)	0.0356 (7)	−0.0038 (7)	−0.0100 (7)	0.0035 (6)
C10	0.0392 (7)	0.0362 (7)	0.0335 (7)	−0.0055 (5)	−0.0056 (5)	−0.0007 (5)
C11	0.0356 (7)	0.0445 (8)	0.0385 (7)	−0.0007 (6)	−0.0070 (6)	0.0028 (6)
N1	0.0290 (6)	0.0456 (6)	0.0328 (6)	−0.0039 (5)	−0.0047 (4)	0.0009 (5)
O1	0.0320 (6)	0.0977 (10)	0.0558 (7)	−0.0034 (6)	0.0003 (5)	0.0010 (7)
S1	0.0463 (3)	0.0700 (3)	0.0387 (2)	−0.00116 (19)	0.00624 (17)	−0.00050 (18)

### Geometric parameters (Å, °)

**Table:**

C2—O1	1.2244 (19)	C6—H6B	0.9700
C2—N1	1.3494 (19)	C6—H6A	0.9700
C2—C3	1.509 (2)	C7—C10	1.364 (2)
C3—C4	1.531 (2)	C7—S1	1.7216 (15)
C3—H3B	0.9700	C8—C9	1.359 (3)
C3—H3A	0.9700	C8—S1	1.7127 (19)
C4—C5	1.530 (2)	C8—H8A	0.9300
C4—H4B	0.9700	C9—C10	1.4271 (19)
C4—H4A	0.9700	C9—H9A	0.9300
C5—N1	1.4730 (18)	C10—C11	1.4983 (19)
C5—C6	1.506 (2)	C11—N1	1.4530 (18)
C5—H5A	0.9800	C11—H11B	0.9700
C6—C7	1.498 (2)	C11—H11A	0.9700
			
O1—C2—N1	124.92 (15)	C5—C6—H6A	109.9
O1—C2—C3	126.64 (15)	H6B—C6—H6A	108.3
N1—C2—C3	108.44 (13)	C10—C7—C6	124.86 (13)
C2—C3—C4	104.44 (13)	C10—C7—S1	111.24 (11)
C2—C3—H3B	110.9	C6—C7—S1	123.89 (11)
C4—C3—H3B	110.9	C9—C8—S1	111.60 (12)
C2—C3—H3A	110.9	C9—C8—H8A	124.2
C4—C3—H3A	110.9	S1—C8—H8A	124.2
H3B—C3—H3A	108.9	C8—C9—C10	112.69 (15)
C5—C4—C3	103.88 (13)	C8—C9—H9A	123.7
C5—C4—H4B	111.0	C10—C9—H9A	123.7
C3—C4—H4B	111.0	C7—C10—C9	112.41 (14)
C5—C4—H4A	111.0	C7—C10—C11	122.46 (13)
C3—C4—H4A	111.0	C9—C10—C11	125.11 (13)
H4B—C4—H4A	109.0	N1—C11—C10	110.65 (12)
N1—C5—C6	111.65 (12)	N1—C11—H11B	109.5
N1—C5—C4	102.22 (12)	C10—C11—H11B	109.5
C6—C5—C4	115.73 (14)	N1—C11—H11A	109.5
N1—C5—H5A	109.0	C10—C11—H11A	109.5
C6—C5—H5A	109.0	H11B—C11—H11A	108.1
C4—C5—H5A	109.0	C2—N1—C11	122.02 (12)
C7—C6—C5	109.13 (12)	C2—N1—C5	112.77 (12)
C7—C6—H6B	109.9	C11—N1—C5	121.72 (12)
C5—C6—H6B	109.9	C8—S1—C7	92.04 (8)
C7—C6—H6A	109.9		
			
O1—C2—C3—C4	171.40 (18)	C7—C10—C11—N1	−3.3 (2)
N1—C2—C3—C4	−9.31 (19)	C9—C10—C11—N1	178.30 (14)
C2—C3—C4—C5	23.40 (19)	O1—C2—N1—C11	10.3 (3)
C3—C4—C5—N1	−28.18 (17)	C3—C2—N1—C11	−168.98 (14)
C3—C4—C5—C6	−149.74 (14)	O1—C2—N1—C5	169.56 (17)
N1—C5—C6—C7	44.61 (18)	C3—C2—N1—C5	−9.74 (18)
C4—C5—C6—C7	160.97 (13)	C10—C11—N1—C2	−173.84 (13)
C5—C6—C7—C10	−24.2 (2)	C10—C11—N1—C5	28.76 (19)
C5—C6—C7—S1	156.95 (12)	C6—C5—N1—C2	148.74 (14)
S1—C8—C9—C10	−0.2 (2)	C4—C5—N1—C2	24.42 (17)
C6—C7—C10—C9	−178.47 (14)	C6—C5—N1—C11	−51.94 (19)
S1—C7—C10—C9	0.54 (17)	C4—C5—N1—C11	−176.27 (13)
C6—C7—C10—C11	2.9 (2)	C9—C8—S1—C7	0.39 (15)
S1—C7—C10—C11	−178.07 (11)	C10—C7—S1—C8	−0.53 (13)
C8—C9—C10—C7	−0.3 (2)	C6—C7—S1—C8	178.49 (15)
C8—C9—C10—C11	178.31 (15)		

### Hydrogen-bond geometry (Å, °)

**Table:**

*D*—H···*A*	*D*—H	H···*A*	*D*···*A*	*D*—H···*A*
C9—H9A···O1^i^	0.93	2.60	3.379 (2)	142

Symmetry codes: (i) −*x*, −*y*+2, −*z*+2.
